# A Systematic Review and Meta-Analysis of Heart Rate Variability in Assessing Surgeons’ Stress Levels

**DOI:** 10.3390/healthcare14040484

**Published:** 2026-02-13

**Authors:** Abdulaziz Almuhini, Zeeshan Raza, Rossana Castaldo, Silvio Pagliara, Leandro Pecchia

**Affiliations:** 1 Department of Biomedical Technology, College of Applied Medical Sciences in Al-Kharj, Prince Sattam bin Abdulaziz University, Al-Kharj 11942, Saudi Arabia; 2 Applied Biomedical Signal Processing and Intelligent eHealth (ABSPIE) Lab., School of Engineering, University of Warwick, Coventry CV4 7AL, UK; zeeshan-raza.raza@warwick.ac.uk (Z.R.); rossana.castaldo1990@gmail.com (R.C.); silviom.pagliara@unica.it (S.P.); leandro.pecchia@unicampus.it (L.P.); 3 Department of Colorectal Surgery, University Hospital Coventry and Warwickshire, Coventry CV2 2DX, UK; 4 IRCCS Synlab SDN, 80143 Naples, Italy; 5 Department of Letters, Languages and Cultural Heritage, University of Cagliari, 09124 Sardinia, Italy; 6 School of Engineering, Università Campus Bio-Medico di Roma, Via Álvaro del Portillo, 00128 Rome, Italy

**Keywords:** HRV, heart rate variability, intraoperative stress, surgeons, robotic surgery, laparoscopic surgery

## Abstract

**Background**: Surgeons’ stress can significantly impact performance, leading to medical errors. Various factors contribute to stress, including a procedure’s complexity and surgeon experience. However, the field currently lacks a standardised approach to measuring stress in surgeons of different ages and experience levels. **Materials and Methods:** This systematic review evaluated heart rate variability (HRV) measures used in surgery to assess stress, considering surgeon age and experience. We searched PubMed, Scopus, and Web of Science following PRISMA guidelines, focusing on studies reporting HRV measurements in surgeons during surgery and comparing different surgeries, procedures, or surgeon experiences. **Results:** Out of 1821 reviewed studies, nine papers met the criteria, which involved 74 subjects. These studies reported various HRV measures, including heart rate, RMSSD, SDNN, pNN50, LF/HF ratio, LF, and HF. Although all time-domain features tended to show a negative response to stress, frequency-domain measures exhibited consistent patterns. However, these findings should be considered preliminary due to the small number of papers, high heterogeneity among studies, and the fact that no study has established a standard for comparing HRV across different surgeon ages or experience levels. **Conclusions**: Finally, these findings call for future studies with robust designs to explore the use of HRV parameters for measuring stress over time while considering surgeon age and experience.

## 1. Introduction

Stress has been dubbed the health epidemic of the 21st century by the World Health Organisation (WHO) [1]. Numerous studies show that job stress has remained the leading source of stress for adults over the past few decades [2,3]. This holds true for medical professions as well, where there is growing concern regarding increasing stress among medical students, trainee doctors, and consultants in medicine and surgery alike [4,5,6,7,8]. It has also been linked to the causation and/or exacerbation of disease in most organ systems of the body, including increased rates of heart attack, hypertension, obesity, addiction, anxiety, depression, and other mental disorders [9,10].

However, there are other factors that can affect a surgeon’s ability to manage stress during different surgical procedures, including age and experience [11]. With more experience and expertise, a surgeon may develop better coping mechanisms and a greater sense of control, which can help reduce stress and improve performance during surgery. On the other hand, younger or less experienced surgeons may have higher levels of stress and anxiety, which can impact their performance. Additionally, as surgeons age, they may experience physical changes that can affect their ability to perform surgery, such as a decrease in fine motor skills and dexterity, potentially increasing stress levels.

Stress can be defined as a “state of threatened homeostasis or disharmony” that occurs when an individual is emotionally activated and perceives that their cognitive and/or performance requirements outweigh the available resources [12]. Furthermore, a review that examined the concept of stress suggested that stress in a particular situation mainly depends on the controllability and predictability of that situation, as both influence the response [13]. As a result, an adaptive stress response occurs under allostasis.

Stress in medical practice has long been a topical subject. It is a highly personalised phenomenon that varies between individuals, depending on individual vulnerability and resilience. The British Medical Association (BMA) published a report on stress in junior and senior doctors, revealing that significant levels of stress existed in this cohort as a result of factors such as peer pressure, social expectation, training, and a hostile job environment [12].

Surgeons work in a stressful environment due to performing technically challenging surgeries under time constraints [13,14]. This has a direct impact on surgical performance and patient safety [15,16]. Moreover, surgical teams frequently encounter highly complex crisis situations that can cause considerable stress, directly contributing to adverse patient outcomes [17,18]. There is a growing concern regarding different acute and chronic stressors in surgeons leading to inferior surgical performance, quality of care, and patient outcomes [19,20,21]. Long-term exposure to stress has been linked to errors in decision-making and burnout [22].

A recently published study found that committing an error or even being under the pressure of not committing an error can activate the ANS response, which is reflected in HRV as a result [23]. Surgeons perceived mental health treatment as being necessary due to the advancing nature of surgical techniques; surgeons will continue to experience increasing stress, thus making the stress assessment more pertinent and essential than before. In fact, mental stress raises concerns about the quality of work delivered by healthcare professionals, since it is directly linked to reducing their performance [24]. Surgeons perceived mental health treatment as being punitive, which may have discouraged them from seeking such treatment [25].

Traditionally, objective assessments of acute stress are carried out with heart rate and HRV, as these measures are widely validated indicators of autonomic nervous system reactivity. Moreover, they have been used extensively in psychophysiological research to quantify stress responses in real time [26,27,28]. HRV in particular is considered advantageous compared with other non-invasive measures, such as electrodermal activity or salivary biomarkers, because it can offer a high temporal resolution, reflects both sympathetic and parasympathetic modulation, and can be continuously monitored without disrupting workflow, especially in medical professions [29,30]. The most well-studied stress biomarker is cortisol, but others, such as alpha-amylase, secretory immunoglobulin A, and chromogranin A, have also been associated with the stressed state [31,32,33,34].

Given the nature of a surgeon’s work, it is preferable to avoid conducting saliva tests multiple times during a stressful surgery, as interrupting the surgeon to collect repeated samples may introduce an external stressor that could influence the measurement of stress. Therefore, considering the nature of a surgeon’s work, investigating the stress levels in surgeons through non-invasive tools such as HRV is preferable.

In clinical terms, HRV refers to beat-to-beat variations in the heart rate or the duration of the R-R interval [35,36,37]. For some researchers, it is defined as the heart–brain interaction, as it reflects the brain’s response to daily stimuli [38].

HRV has three main components: time domain, frequency domain, and non-linear domain. The time domain looks at how much the time between heartbeats varies; the frequency domain looks at how power is spread across different frequency ranges (high, low, very low, and ultra-low); and the non-linear features help to understand how unpredictable a time series is when using these methods [28].

Over the years, several systematic literature reviews have been published aiming to link HRV to stress, inflammation, cognitive function, and myocardial infarction [27,39,40,41]. To the best of the authors’ knowledge, none of these reviews covered HRV and its relationship with mental stress in surgeons or attempted to make comparisons among various surgical procedures and different levels of experience in this context, which highlights a gap in the literature. However, a meta-analysis conducted by Kim et al. supports the use of HRV for the objective assessment of stress [27].

In fact, HRV can be used to monitor surgeons by assessing mental stress and helping to maintain their physiological stability, as shown by AF et al. in a systematic review focusing on surgeons. However, this review did not differentiate between different surgical procedures and different levels of experience [42]. HRV can vary depending on the type of surgery being performed and the surgeon’s experience, expertise, and skill. Some studies have shown that HRV can be used to predict the physiological response of surgeons to different surgical procedures, including their stress levels, workload, and mental workload [27].

Moreover, abnormal HRV can lead to serious mental disorders due to the nature of the body-to-brain balance [43]. Overall, monitoring HRV in surgeons during surgery can offer useful information regarding their physiological status and help maintain their well-being during demanding procedures. HRV signals and the metrics derived from them have been used in both simulated and real surgical settings [44,45]. This methodology involves measuring stress in the operating room in response to acute environmental stressors.

This systematic literature review was conducted to determine the influence of stress on surgeons during surgical procedures and to correlate the level of stress with their age and experience, considering the heterogeneity of different surgeries under stressful and non-stressful conditions. The added value of this review is that, to our knowledge, it is the first to comprehensively synthesise HRV-based stress assessment specifically in relation to surgeon demographics. This work identifies a lack of standardised HRV protocols across studies, underscoring the need for consistent measurement frameworks. The main learning points are that HRV might reliably reflect intraoperative stress, but methodological variability limits comparability, and future studies should adopt standardised protocols to improve assessment and intervention development. However, this systematic review is not using or relying on AI, and it has been declared in compliance with TITAN guidelines [46].

## 2. Materials and Methods

In order to ensure transparency and ensure that our review adhered to a predefined protocol, thus reducing the risk of bias and duplication of research efforts, this systematic review was registered with PROSPERO (registration number: CRD42022326732), the International Prospective Register of Systematic Reviews, and no divisions were made since then. Also, it has been reported to be in line with the Assessing the Methodological Quality of Systematic Reviews (AMSTAR) Guidelines [47].

In detail, this systematic literature review was conducted to identify the differences in HRV parameters in surgeons and investigate possible associations with age/experience to determine how stress can affect their performance. This study followed the PRISMA (Preferred Reporting Items for Systematic Reviews and Meta-Analyses) statement [48]. The purpose of this type of review is to achieve the highest level of reliable results.

### 2.1. Search Strategy

In this review, the Patient/Problem, Intervention, Comparison, and Outcome (PICO) search strategy was used to identify and select studies [49]. Potentially relevant studies were identified by searching PubMed, Scopus, and Web of Science electronic databases published from January 2005 to April 2025.

Further, a combination of Boolean operators (e.g., [(“mental stress” OR “mental fatigue” OR “occupational stress” OR “physiological stress”) AND (“wearable device” OR “continuous monitoring”) AND “Surgery” OR “Surgical robotics” OR “Laparoscopic surgery” OR “operation room” OR “robotic surgery”)]) was used in the string to retrieve the related papers (see Supplementary File S1 for full used string).

### 2.2. Eligibility Criteria

The papers were screened and the data were extracted and analysed. The inclusion criteria included the following: studies in English, peer-reviewed papers, intervention studies focused on measuring HRV for surgeons, and differentiation between at least two kinds of surgeries or surgical procedures. Finally, the data were extracted, in accordance with PRISMA, and titles and abstracts of studies were retrieved. Then, a linear reference search was conducted by checking the references of the studies identified in the search. Two researchers, who were blinded to the author information of the articles, independently screened all identified records for inclusion. In case of disagreements, a third author was consulted.

After the initial screening of the titles and abstracts, papers published before April 2025 were considered suitable for this review after meeting the previously mentioned criteria.

The exclusion criteria were defined as follows: (1) studies conducted before 2005, (2) lack of reference to surgeons or surgical settings, (3) not published in a journal article, (4) not reporting at least three of the HRV features, and (5) not reporting either age or experience.

Studies reporting fewer than three HRV parameters were excluded because limited reporting restricted meaningful comparison and consistent synthesis across autonomic domains. Including studies with only a single HRV metric would have constrained interpretation of stress-related responses. We acknowledge that this decision may have reduced the number of eligible studies and excluded potentially relevant evidence. All included studies focused on measuring HRV in surgeons during the surgery as well as comparing the acquired readings between two or more age groups or two or more experienced groups. Additionally, the studies focused on comparing the different types of surgical procedures involved. However, to broaden this study, no limitations were applied to sample size, level of experience, type of surgery, surgical speciality, or type of surgical procedure. This variance represents a major source of heterogeneity.

### 2.3. Data Extraction

We extracted, where possible, the following data from the studies: authors, title, year of publication, availability of original data, analysis of HRV measures (time, frequency, or non-linear), participant characteristics (age, sex, and experience), study design, and type of surgical procedures performed. Data extraction was independently performed by two reviewers and subsequently cross-checked to ensure accuracy and consistency. We summarised the initial findings for each study, including the level of experience for the recruited participant, the type of surgeries performed, and the HRV components used for the assessment of stress. In cases of missing data, one of the authors [AA] contacted the paper’s authors for the required information.

### 2.4. Certainty Assessment

The Mixed Methods Appraisal Tool (MMAT) was used to assess the risk of bias in the included trials. Since the MMAT is applicable for observational and randomised case studies and is designed for systematic reviews that include different types of studies, it was appropriately used here [50]. The MMAT contains a sequence of screening questions to be answered, and the score reflects the overall judgement, where a smaller number shows minor methodological limitations. This assessment was used to inform the interpretation of findings rather than to exclude studies.

### 2.5. Data Synthesis

This review investigated studies that reported heart rate variability, heart rate, frequency-domain (LF, HF, LF/HF ratio), time-domain (standard deviation (SD), and root mean square (RMSSD) parameters. Due to expected clinical and methodological heterogeneity—including differences in HRV acquisition methods, baseline definitions, surgical procedures, and participant characteristics—results were not pooled when studies were deemed too heterogeneous. In such cases, findings were summarised narratively and presented in tables or figures. Sources of heterogeneity were considered when interpreting pooled estimates, and subgroup analyses were conducted where appropriate to improve comparability. The purpose of pooling was not to produce definitive effect sizes, but to explore directional consistency and quantify the degree of between-study variability.

### 2.6. Meta-Analysis

Considering the low number of included studies, an exploratory meta-analysis was performed; the included studies were extensively examined to observe results. HRV measures were pooled if reported in more than three papers. When shortlisted papers for meta-analysis reported features in a non-conventional way, these measures were either excluded from the pooling or converted, if possible.

When the papers did not report HRV feature values such as means and standard deviations, they were transformed to estimate the mean from the given median, lowest value, and highest value by using the equation suggested by Hozo et al. The sample size was used to approximate the mean, *x* [51]. Then, to validate the robustness of the calculation, the approach suggested by Wan et al. was applied and the result of both were constant [52]. This agreement suggested minimal bias due to potential skew.

*m* = Median;

*a* = The smallest value (minimum);

*b* = The largest value (maximum);

(1) $$x\approx \frac{a+2m+b}{4}$$

In this paper, standard techniques for systematic reviews with meta-analyses were used to pool the HRV measures: mean difference (MD) with 95% confidence intervals (95% CIs) and *p*-values (*p*). According to the Q statistic’s assessment of the heterogeneity across the studies, a random effect model was employed. Complementing the Q test, we also computed the I^2^ statistic, which provides an index of the level of heterogeneity across the studies. Specifically, I^2^ indicates the proportion of total effect size variability attributable to differences between studies, as opposed to sampling error within studies. If the *p*-value was less than 0.05, the differences and 95% CI were deemed significant. These statistics were calculated using the RevMan 5.4 Software utility.

In the first step, the included studies were added to the programme, and our comparisons and their outcomes were applied to the entered data. However, some studies did not report all the parameters: for example, Rieger et al. did not report the mean of the surgeon’s age and rather provided the median, minimum, and maximum age values. As a result, the equation mentioned in Hozo et al. was applied to the known values to find the mean of the ages, and the result of this was used for the main tests conducted in the review manager program [51,53]. In the case of high heterogeneity, subgroup analysis was performed in order to find out the reasons behind this.

## 3. Results

### 3.1. Study Selection

**Figure 1:**
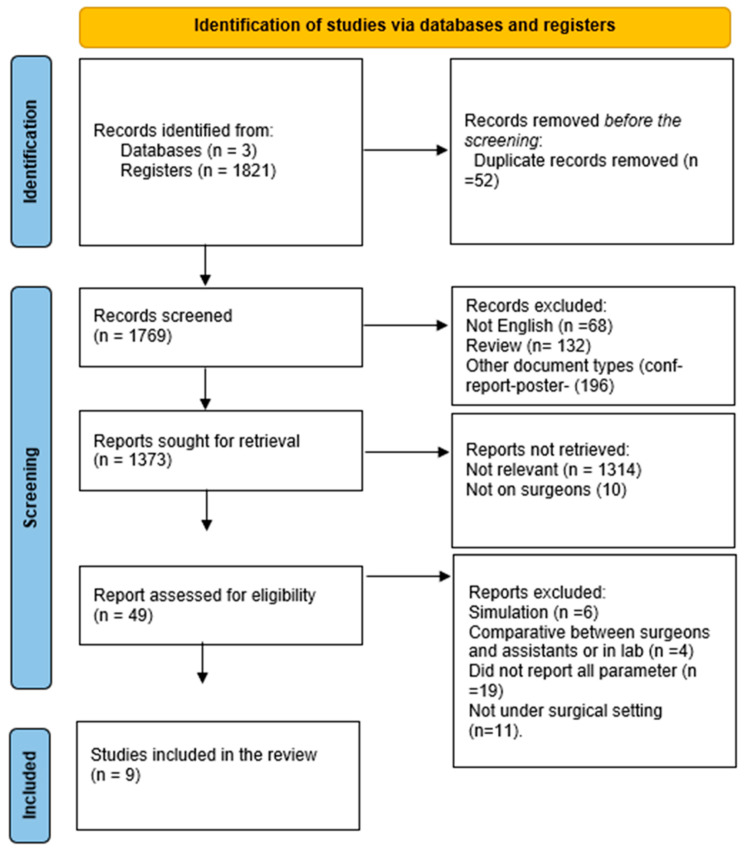
The PRISMA flow chart illustrates the method and criteria used in this review selection process.

The relevant papers in PubMed, Scopus, and Web of Science were published within the last 20 years, from 2005 until April 2025. Altogether, the three databases searched revealed 1821 papers. After the removal of duplicates, the titles and abstracts were screened to determine if they were suitable based on the inclusion criteria. A total of 30 studies were considered potentially relevant to the review question after the screening process was completed. Eventually, full-text screening was carried out on nine studies out of 1821, which included 74 subjects to be tested (Figure 1). The string was first developed in PubMed and then adapted for Scopus and Web of Science.

### 3.2. Study Characteristics

In total, 74 participants were involved across the nine studies. From these, 13 (17.6%) were women, 38 (51.4%) were men, and 23 (31%) did not report gender. The sample size varied from having a single participant to as many as 20 in several of the included studies, with an average of eight participants per study. Regarding age, 41 participants (55.4%) were between 30 and 40 years, 19 (25.6%) were between 40 and 50 years, and 3 (4.1%) were over 50 years, while 11 (14.9%) did not report age.

With regard to experience, several studies differentiated between experienced and less experienced surgeons (e.g., Heemskerk et al. [45], Rieger et al. [53], Klein et al. [54], and Haffar et al. [55]). Weenk et al. [56] investigated consultants, senior residents, and junior residents, whereas Cap et al. [57] investigated surgeons based on the number of surgeries they had completed in order to show their experience. Finally, Jones et al. [58] did not mention the individual experience of the consultant colorectal surgeons, while Kwon et al. [59] divided the surgeons into three groups based on experience: <5 years, 5–15 years, and >15 years. This extreme variation in reporting the experience level made it hard to find a common ground among all the papers. To enable a consistent classification for synthesis purposes, we adopted pragmatic operational definition of expertise as performing at least 1000 surgeries or having more than 10 years of clinical practice, which is consistent with thresholds cited in the surgical training literature [60,61]. However, we acknowledge that this definition is not universally standardised and that the lack of uniform reporting in the primary studies represents a limitation that may affect the precision and generalisability of the findings. Based on this operational classification, 48 (64.9%) expert surgeons and 23 (31.1%) non-expert surgeons were found, and four (5.4%) were inconclusive. This indicates that two-thirds of the sample were experts and almost a third were non-experts. Table 1 shows the full details for each study.

### 3.3. Quality Assessment

MMAT was used to assess the risk of bias in the included trials [50]. This resulted in two qualitative studies, one non-randomised study, and one mixed-methods study, while the remaining five studies were found to be quantitative in nature, as shown in Table 2. Most studies met MMAT criteria overall; however, some showed methodological uncertainty in specific domains. In particular, “Can’t tell” ratings were frequently assigned for risk of nonresponse bias, appropriateness of statistical analysis, and whether confounders were adequately accounted for in the design or analysis. These uncertainties reflect limitations in reporting and potential residual bias (see Supplementary File S2 for a full detailed assessment).

### 3.4. Time-Domain Features

The domain features reported in the included studies were the HR, RMSSD, SDNN, and pNN50. However, HR, RMSSD, and SDNN were reported in four out of nine studies. pNN50 was reported in two out of the nine studies, as shown in Table 3. Apart from HR in Cap et al. [57] and Haffar et al. [55], all of the time-domain features tended to act with the same pattern, which reflected stress under surgical settings. (The full measures are detailed in Supplementary File S1 Tables S1–S4).

### 3.5. Frequency-Domain Features

Regarding the frequency-domain features, the reported measures were LF, HF, and LF/HF ratio. Specifically, LF and HF were reported in two out of nine studies. In contrast, there were three studies that reported LF/HF ratio, as shown in Table 4. Similarly to the time domain, the same pattern in response to stress was shown here as well. (The full measures are detailed in Supplementary File S1 Tables S5–S7).

### 3.6. Meta-Analysis

HRV features were pooled only if reported in more than three papers. The RevMan review manager was used to perform the data analysis. Further, meta-analysis (MD) with a 95% confidence interval (95% CI) and the random effect model were used. Some of the studies reported the readings for some of the parameters in terms of mean and standard deviation, such as those of Weenk et al. [56] and Kwon et al. [59]; some of them were reported as the median, lowest value, and highest value, such as in Klein et al. [21], Rieger et al. [53], and Weenk et al. [56], which facilitated calculation of the approximate mean based on Hozo et al.’s [51] suggested calculation; and moreover, some of the studies gave detailed data and readings, such as Cap et al. and Kwon et al. They did not report direct readings other than analysis of variance [57,59]. Therefore, despite making an unsuccessful attempt to contact the authors for the raw data, the means for some parameters were obtained in certain studies through the previous clarification.

**Figure 2:**
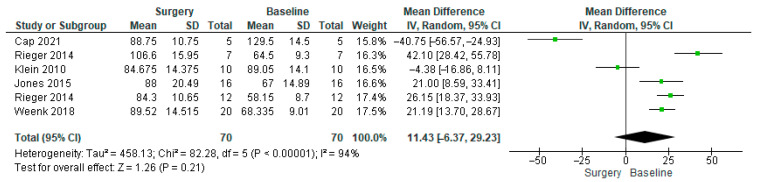
Heart rate forest plot, including all of the studies reporting it [53,56,57,58,62].

**Figure 3:**
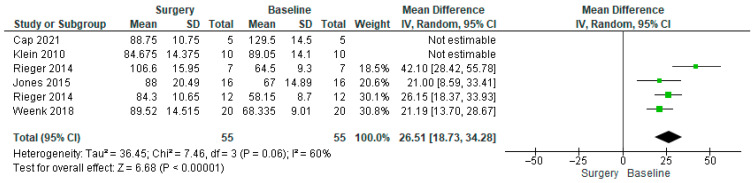
Heart rate forest plot of subgroup analysis, excluding Klein et al. and Cap et al. [53,56,57,58,62].

The heart rate forest plot (Figure 2) indicated high heterogeneity, which necessitated conducting a subgroup analysis. In the subgroup analysis (Figure 3), Klein et al. and Cap et al. were excluded to improve methodological consistency across the included data [54,57]. While this approach was useful for individual-level comparisons, it limited the interpretability of pooled results using raw heart rate values. Excluding these two studies helped ensure that the meta-analysis focused on comparable measures and conditions across studies (Table 3).

**Figure 4:**
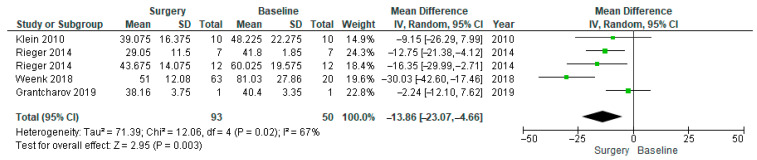
SDNN forest plot, including all of the studies reporting it [53,56,62,63].

**Figure 5:**
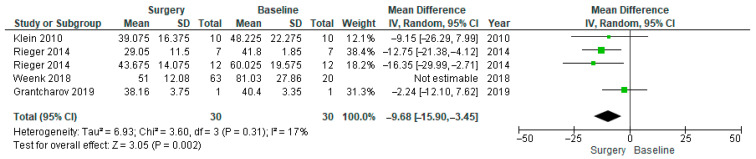
SDNN forest plot of subgroup analysis, excluding Weenk et al. [53,56,62,63].

Also, in the SDNN forest plot (Figure 4), the heterogeneity was high, which led us to conduct a subgroup analysis for the SDNN. Subgroup analysis was performed (Figure 5), and Weenk et al. was excluded from the subgroup analysis due to methodological differences that likely contributed to increased heterogeneity [56]. Removing this mismatch likely explained the larger observed effect and the substantial drop in heterogeneity (from 67% to 17%) after its exclusion.

Overall, these findings highlight the variability in HRV reporting and the challenges of standardising stress measurement in surgical settings. The pooled HRV data suggest the potential of using HRV metrics to assess surgical stress across different experience levels, but further studies are needed to address inconsistencies in measurement and reporting methods.

## 4. Discussion

A systematic search of PubMed, Scopus, and Web of Science identified 1821 papers published between 2005 and April 2025. After screening for duplicates and relevance, nine studies with 74 participants were included. These studies varied in size and participant age, with most subjects aged 30–40 years and including both experienced and less experienced surgeons. The studies were evaluated using the MMAT, revealing a mix of quantitative and some mixed-methods research. Some studies had unclear reporting on nonresponse bias, statistical methods, and confounder control, which introduced uncertainty into effect estimates and may have contributed to heterogeneity. These limitations reduced confidence that observed HRV differences reflected stress-specific mechanisms, so findings were interpreted cautiously. The HRV measures reported included time-domain features like HR, RMSSD, SDNN, and pNN50, with frequency-domain features such as LF, HF, and LF/HF ratio reported less frequently.

The exploratory meta-analysis pooled HRV features were reported in more than three studies, showing significant variability across studies in terms of design, baseline measures, and surgeon experience. Heart rate was the most commonly reported HRV measure, and pNN50, LF, and HF were reported less frequently. Variability in baseline heart rate measurements and study designs contributed to heterogeneity, underlining the need for standardised methods to assess HRV in surgical environments.

The meta-analysis revealed substantial variation in how HRV parameters were reported and analysed, making direct comparisons difficult. Moreover, the calculated mean in one of the studies might have contributed to increasing the heterogeneity. However, to address this, time-domain metrics—specifically heart rate and SDNN—were analysed separately, and subgroup analyses were conducted to improve consistency.

For heart rate, the full pooled analysis (Figure 2) showed high heterogeneity (I^2^ = 94%) and a non-significant overall effect (MD = 11.54, 95% CI [−5.00, 28.09], *p* = 0.17). In the subgroup analysis (Figure 3), Klein et al. and Cap et al. were excluded due to methodological inconsistencies. Klein et al. compared two surgical techniques rather than surgery versus baseline, while Cap et al. used a calculated normalised heart rate metric that limited comparability [54,57]. Their exclusion reduced heterogeneity to 60% and revealed a significant increase in heart rate during surgery (MD = 26.51, 95% CI [18.73, 34.28], *p* < 0.00001).

A similar pattern was observed with SDNN. The full analysis (Figure 4) indicated moderate-to-high heterogeneity (I^2^ = 67%) and a significant reduction in SDNN during surgery (MD = −13.86, 95% CI [−23.07, −4.66], *p* = 0.003). In the subgroup analysis (Figure 5), Weenk et al.’s study was excluded due to a mismatch between the baseline which was recorded once per participant (n = 20) and the surgical conditions which were measured repeatedly across various procedures (n = 63) [56]. This exclusion reduced heterogeneity to 17%, with the effect remaining statistically significant (MD = −9.68, 95% CI [−15.90, −3.45], *p* = 0.002).

Interpretation of the pooled heart rate and SDNN findings should consider that age and sex were inconsistently reported and often skewed toward predominantly male, mid-career samples (e.g., Klein et al.; Rieger et al.; and Jones et al.), despite known age- and sex-related influences on autonomic regulation [53,54,58]. Surgical context also varied widely across studies (e.g., laparoscopic procedures in Klein et al., colorectal surgery in Jones et al., and orthopaedic procedures in Kwon et al.), which may have independently affected heart rate and SDNN [54,58,59]. In addition, several studies included multiple procedures per surgeon (e.g., Grantcharov et al. and Rieger et al.) without accounting for within-surgeon clustering, potentially inflating precision [53,63]. These factors meant that some observed differences may have reflected demographic composition, procedural mix, or repeated-measures dependence rather than stress alone.

Although statistically significant changes in heart rate and SDNN were observed in the analyses, the clinical or practical significance of these differences remain uncertain. Changes like this may simply reflect normal physiological responses to posture, movement, or sustained concentration, all of which occur during surgery. Therefore, HRV changes cannot be assumed to reflect psychological stress alone without considering concurrent physical and environmental influences. HRV is a sensitive but non-specific marker of autonomic activity, and in the complex surgical setting it may reflect a combination of cognitive load, physical exertion, circadian factors, and emotional stress. As such, HRV should not be interpreted as a standalone indicator of psychological stress.

These findings suggest that while time-domain HRV metrics appear responsive to surgical stress, their interpretability is strongly influenced by inconsistencies in study design, baseline definitions, and data reporting. The lack of sufficient frequency-domain and non-linear data precluded meta-analysis in these domains. Standardised methodologies and clearer reporting practices are needed to improve the reliability and clinical value of future HRV research in surgical contexts. However, since expertise in some studies was classified using a pragmatic criterion based on procedural volume, distinctions between “experienced” and “less experienced” groups should be read with caution. Surgical proficiency is contingent upon case complexity, technical acumen, decision-making capabilities, and non-technical skills, which were not uniformly documented across investigations.

Previous studies have explored the link between HRV and stress in surgeons, but there are still significant gaps, especially when it comes to how stress levels may differ based on a surgeon’s age, experience, or the specific type of surgery being performed. Although HRV is commonly used to measure stress in various fields, its application in surgery has been inconsistent, largely due to differences in the HRV measures used and the methods for assessing stress itself [64]. Moreover, previous research demonstrating that HRV consistently reflects acute stress responses aligns with the findings [26,27]. However, unlike prior reviews primarily focusing on healthcare workers in general, our synthesis highlights a unique gap in standardised HRV protocols within surgical environments. While earlier studies proposed HRV as a promising stress biomarker, this analysis underscores significant barriers to generalisability due to methodological inconsistency, highlighting the need for harmonised study designs.

This study builds on previous research by taking a broader look at how HRV responds across different surgical settings and among surgeons of varying experience levels. While past studies have focused on only a limited set of HRV parameters or examined just one type of surgery, the analysis in this study pulls together multiple HRV features, including both time-domain and frequency-domain measures, and explores how they relate to the surgeon’s experience. By differentiating between expert and non-expert surgeons, it offers a preliminary insight into how HRV may reflect stress, although whether differences represent physiological adaptation over time cannot be determined from the available data.

This study is unique, as it synthesises findings from studies with conflicting results. By doing so, it highlights the need for consistent measurement methods, something that earlier studies, such as those by Weenk et al. and Rieger et al., pointed out but could not fully resolve due to variations in design [53,56]. This work provides a clearer path for future research, offering practical insights into how we can improve the reliability of HRV measurements in surgical settings.

This study shows that HRV could be a valuable tool for monitoring stress in surgeons, which could have a significant impact on both their performance and patient safety [65]. The findings suggest that measures like heart rate and SDNN are effective for assessing stress during surgery. Using HRV in real-time could alert surgeons to high stress levels, enabling quick interventions to reduce stress and prevent medical errors. HRV could also complement other tools used to measure stress, providing a non-invasive way to track how stress affects surgeons while they perform surgery [66].

Although HRV has been explored as a marker of physiological stress, current evidence is limited, heterogeneous, and insufficient to support routine or real-time intraoperative use. Most studies are small, observational, and use varied acquisition and analysis methods, limiting comparability and clinical translation. Feasibility and ethical challenges also remain. Future research should evaluate minimally intrusive wearable sensors in real operating rooms, improve automated signal processing, and determine how HRV data can be meaningfully summarised or fed back without disrupting workflow [67]. Establishing physiologically relevant stress thresholds and clear frameworks for data privacy and governance is also essential [68,69]. Multi-centre prospective studies, greater consensus on HRV measurement and reporting protocols (e.g., baseline definitions, recording duration, and core metrics), and integration with psychological, hormonal, and other physiological stress markers are needed to strengthen validity. Patient complexity may also be an important factor linking operative demands to physiological stress responses [70]. At present, no clinically meaningful intraoperative HRV thresholds are established. Reported differences are mostly group-level and method-dependent, and it remains unclear what magnitude of change would indicate excessive stress or impaired performance. Therefore, current evidence remains exploratory and does not support actionable HRV cut-offs for intraoperative monitoring.

This review lays the groundwork for future research into HRV as a tool to monitor stress in surgical environments. However, there are still a few gaps to address. For example, more work is needed to standardise HRV measurement techniques and to include a broader range of surgeries and surgeon experience levels [71]. Future studies could also explore the use of HRV alongside other stress indicators, like cortisol, to provide a more comprehensive understanding of stress during surgery. Long-term studies could also help us understand the lasting impact of stress on surgeons’ health and how HRV could be used to track stress over time [66,72].

Chronic intraoperative stress may contribute to longer-term autonomic changes, including sustained sympathetic activation and reduced parasympathetic tone, leading to lower baseline HRV over time [26]. Prolonged stress has also been proposed to affect hypothalamic–pituitary–adrenal axis function and cortisol regulation [68]. These processes are commonly described within the framework of allostatic load, the cumulative physiological burden of repeated stress, which has been associated with reduced HRV [69].

Ultimately, the hope is to see HRV-based stress monitoring used in everyday surgical practice. The goal would be to develop a system that provides real-time feedback to surgeons about their stress levels, pending further validation. If validated, this could help surgeons manage stress better, leading to fewer medical errors and better outcomes for patients. Over time, if its benefits are proven, HRV could potentially become part of surgical care, improving not just the well-being of surgeons but also the overall quality of care in the operating room.

However, there are some challenges that need to be overcome before HRV can be widely used in surgery. For one, there is a lot of variation in how HRV is measured across studies, which makes it difficult to create standardised guidelines. Different equipment, data collection methods, and surgeon experience levels can all affect HRV readings. Another issue is figuring out how to use HRV in real time without interfering with the surgical process [73]. Ensuring data are accurate and useful while also being practical in busy operating rooms will require careful planning.

To make real-time HRV monitoring a reality, there is a need for better technology. Wearable devices that measure HRV without interfering with the surgeon’s work will be the key [68]. Along with this, there is a need for advanced data analysis tools that can process HRV data quickly and provide useful insights during surgery. Surgeons will also need to be trained to understand the HRV data and use them in decision-making. For HRV to become a routine part of surgery, collaboration between researchers, clinicians, and tech developers will be essential.

The research into HRV to measure stress in surgeons is both timely and crucial. Surgeons work in high-stress environments, and stress can negatively impact their performance and patient safety. HRV provides an objective, non-invasive method to monitor stress in real-time, offering surgeons a valuable tool to track their stress levels during operations. This area of research is important because it introduces a new approach to understanding and managing stress in the operating room. Not only can it help improve a surgeon’s well-being, it can also enhance patient outcomes by reducing errors that stem from stress-related factors.

The broader importance of this research lies in its potential to improve the sustainability of surgical careers. As surgical techniques become more complex and the pressure on surgeons increases, managing stress is key to maintaining performance over time. This field has the potential to influence both individual surgeons and the healthcare system as a whole, offering a proactive solution to the growing issue of surgeon burnout and fatigue.

These findings should be interpreted considering several methodological limitations. There was considerable variation between studies in terms of HRV measurement approaches, surgical procedures, and sample sizes, making direct comparisons difficult and contributing to heterogeneity. The overall pooled sample was small (74 participants) and had a skewed sex distribution (17.6% female), limiting how well the results reflected the wider surgical workforce. Notably, most studies used cross-sectional designs, which cannot capture the effects of chronic stress and limit conclusions about causal physiological adaptation. The limited and demographically imbalanced sample may therefore have reduced the applicability of these findings across the broader surgical workforce. Sex-related differences in autonomic regulation have been reported, suggesting HRV patterns observed in predominantly male samples may not translate directly to female surgeons. Furthermore, variation in surgical specialities and procedural complexity means stress responses observed in specific contexts may not reflect those in other disciplines.

Reporting of HRV parameters was also inconsistent. Measures such as pNN50, LF, HF, and the LF/HF ratio were available in only a subset of studies, further restricting synthesis. The requirement for multiple HRV parameters may have narrowed the evidence base, as some otherwise relevant studies with partial HRV reporting were excluded. In several cases, “baseline” conditions did not represent true rest but rather work-related states, and clearly defined resting periods were often absent. As a result, some observed differences may have reflected physical workload, posture, or sustained task engagement rather than psychological stress alone, limiting the specificity of HRV as a stress marker. Study quality varied, with many investigations rated as moderate due to incomplete reporting, unclear sampling procedures, and limited control of confounding variables. Definitions of surgeon expertise also differed across studies, adding conceptual variability to subgroup comparisons. In one study, reported means were derived from previously averaged values, representing an additional reporting limitation, although the calculation was verified.

Taken together, these limitations mean that current evidence supports only cautious, preliminary conclusions regarding HRV as a marker of surgical stress and highlights the need for larger, standardised, and methodologically robust studies before firm clinical or practical inferences can be made.

Despite these limitations, this study offers valuable insights. A broad range of studies have been reviewed to provide a clearer picture as to how HRV can be used to monitor stress in surgeons. By examining the most common HRV measures, this study also helps to identify gaps in the current research, which can guide future investigations. This review is particularly important because it lays the groundwork for standardising HRV measurements in surgical settings, a crucial step in making this technology practical for everyday use in operating rooms.

The real value of this research lies in its potential to inform future efforts to translate HRV monitoring into clinical practice. If HRV monitoring can be integrated into the operating room, it could provide real-time feedback to surgeons, helping them to identify when stress levels are rising and take proactive steps to manage them. This could lead to fewer errors, better performance, and an overall reduction in stress-related complications [71].

The ability to monitor HRV could also be extended to surgical training programmes, where future surgeons could learn how to manage stress in a controlled environment. In the long term, HRV could become a routine part of presurgical assessments and intraoperative monitoring, improving not only the health of surgeons but also patient safety [19].

The key challenge is ensuring that HRV monitoring can be used without interfering with surgery. Wearable devices, real-time data analysis, and ease of use are critical factors that require further development [69]. However, if we can overcome these hurdles, HRV monitoring could potentially serve as an adjunctive tool in surgical practice, offering benefits to surgeons and patients.

## 5. Conclusions

This review suggests that surgeons experience physiological stress during many surgical procedures, and heart rate variability (HRV) has been explored as a potential marker of this response. Some HRV indices, including SDNN, RMSSD, LF, HF, and the LF/HF ratio, showed broadly similar trends across studies; however, the evidence was based on a small number of heterogeneous studies with varying methodologies.

Given the limited sample sizes, substantial heterogeneity, and lack of standardised HRV measurement protocols, these findings should be interpreted cautiously. There is currently no consistent framework for comparing HRV responses across surgeons with different levels of experience, specialities, or operative contexts.

Although HRV-based stress monitoring has theoretical appeal, its clinical application remains uncertain. Further research is needed to establish methodological standardisation, validate findings in larger and more representative populations, and define clinically meaningful thresholds. In addition, the feasibility, acceptability, and ethical implications of real-time physiological monitoring in surgical settings require careful evaluation before any consideration of routine use [74,75].

## Figures and Tables

**Table 1:** Evidence table showing a summary of all characteristics in the nine studies.

Authors	Study Design	Number of Surgeons	Surgery Specialty	Number of Surgeries	Experience	Intervention	Assessment of Mental Stress
Klein et al. [62]	Prospective case–control study	Ten consultant surgeons	General surgery	Each surgeon performed 10 laparoscopic cholecystectomies in standard and modern OR (n = 20)	Individual experience->200 laparoscopic cholecystectomy	Continuous heart rate and psychological assessment through questionnaire, VAS score, and BORG CF-12 score	HRV measures: LF, HF, LF/HF, RMSSD, SDNN pNN50
Heemskerk et al. [45]	Observational study	Two consultant general surgeons	General surgery	22 Surgeries: 5 robotic and 2 conventional cholecystectomies while the other performed 6 robotic and 9 conventional cholecystectomies	Exact experience not provided	Continuous ECG recording	HRV measures: Mean HR and R-R intervals VLF, LF, HF, LF/HF ratio
Rieger et al. [53]	Prospective observational study	20 surgeons: Six residents, five fellows, five attending, and four chiefs of medicine	Across different surgical specialities	Continuous ambulatory monitoring over 24 h	Not mentioned explicitly	Ambulatory ECG recording and pre- and post-procedure STAI questionnaire used to divide into stressed and non-stressed groups	HRV measures; SDNN, RMSSD, pNN50, VLF. LF, HF, LF/HF, TP
Weenk et al. [56]	Prospective observational study	20 surgeons: Five consultants, seven fellows and senior residents, and eight junior residents	Across different surgical specialities	Continuous ambulatory monitoring over 48–72 h, 63 elective surgical procedures, and 22 long and 41 short procedures.	Consultants (two or more years of independent practice), fellows (surgeons with less than two years of independent practice), senior residents (postgraduate year [PGY] 5 or 6), and junior residents (PGY 4 or less)	Ambulatory ECG recording and pre- and post-procedure STAI questionnaire	HRV measures; SDNN, RMSSD, VLF. LF, HF, LF/HF,
Cap et al. [57]	Prospective, observational case series.	Five ophthalmic surgeons	Ophthalmology	45 cataract surgeries	Individual experience between 70 and 15,000 surgeries	Continuous heart rate monitoring throughout the procedure	HR and HRV values normalised to the minimal HR and maximal HRV of an overnight baseline measurement, HRV index
Jones et al. [58]	Observational study	Six consultant colorectal surgeons	Colorectal surgery	18 anterior resections	Not mentioned explicitly	Heart rate at 6 predetermined surgical steps and post procedure STAI questionnaire	HRV measures; VLF, LF, HF, LF/HF ratio
Kwon et al. [59]	Prospective observational study	Eight consultant surgeons	Orthopaedic surgery	265 surgeries	3 groups based on experience: <5 years, 5–15 years, >15 years	HRV, 2-channel EEG	HRV measures, BPM, LF/HF ratio EEG measures: Beta 1, 2 and 3 waves
Haffar et al. [55]	Prospective comparative study	One trained arthroplasty surgeon	Orthopaedic surgery	20 robotic total knee arthroplasties, 20 conventional total knee arthroplasties	Experienced in conventional but not robotic	Continuous ECG monitoring throughout the procedure	HR and HRV measures: RMSSD
Grantcharov et al. [63]	Observational study	One consultant general surgeon	Upper gastrointestinal surgery	12 gastric bypass and 7 sleeve gastrectomy surgeries	Not mentioned explicitly	Continuous ECG and surgical performance using the Operating Room Black Box^®^ platform	HRV measures: RMSSD and SDNN

**Table 2:** Type of included papers based on MMAT table.

Authors	Study Type
Klein et al. [62]	Quantitative study
Heemskerk et al. [45]	Non-randomised study
Rieger et al. [53]	Quantitative study
Jones et al. [58]	Mixed method study
Weenk et al. [56]	Qualitative study
Grantcharov et al. [63]	Qualitative study
Cap et al. [57]	Quantitative study
Kwon et al. [59]	Quantitative study
Haffar et al. [55]	Quantitative study

**Table 3:** Summary of the time-domain features.

Time-Domain Features	Authors	Experience (Median Age)	N	Stress		Rest	
				Mean	SD	Mean	SD
Heart rate (Beats/Min)	Klein et al., 2010 [62]	Expert (46)	10	84.675	14.375	89.05	14.1
	Rieger et al., 2014 [53]	Expert (34.5)	12	84.3	10.65	58.15	8.7
	Rieger et al. 2014 [53] *	Expert (39)	7	106.6	15.95	64.5	9.3
	Jones et al., 2015 [58]	Non-expert (N/A)	16	88.0	20.49	67.0	14.89
	Weenk et al., 2018 [56]	Expert and non-expert (N/A)	20	89.52	14.51	68.33	9.01
	Cap et al., 2021 [57]	Expert (40)	2	88.75	10.75	129.5	14.5
	Haffar et al., 2022 [55]	Partially experienced (N/A)	1	81.5	---	90.1	-----
SDNN (ms)	Klein et al., 2010 [62]	Expert (46)	10	39.1	16.4	48.22	22.27
	Rieger et al., 2014 [53]	Expert (34.5)	12	43.7	14.07	60.02	19.57
	Rieger et al., 2014 [53] *	Expert (39)	7	29.05	11.5	41.8	1.85
	Weenk et al., 2018 [56]	Expert and non-expert (N/A)	20	51	12.1	81.03	27.9
	Grantcharov et al., 2019 [63]	Expert(N/A)	1	38.16	3.75	40,4	3.35
pNN50	Klein et al., 2010 [62]	Expert (46)	10	4.52	3.42	7.9	7.15
	Rieger et al., 2014 [53]	Expert (34.5)	12	7.15	4.7	40	16.8
	Rieger et al., 2014 [53] *	Expert (39)	7	5.15	3.7	28.47	3.42
RMSSD (ms)	Klein et al., 2010 [62]	Expert (46)	10	20.67	6.72	22.95	13.5
	Rieger et al., 2014 [53]	Expert (34.5)	12	25.85	8.15	71.9	26.1
	Rieger et al., 2014 [53] *	Expert (39)	7	20.05	7.65	46.75	7.6
	Weenk et al., 2018 [56]	Expert and non-expert (N/A)	20	28.59	10.27	57.9	27.4
	Grantcharov et al., 2019 [63]	Expert (N/A)	1	25.9	---	27.4	---
	Haffar et al., 2022 [55]	Partially experienced	1	14.4	---	12.2	---

* Rieger et al. split his participants into stressed and non-stressed then reported on that basis.

**Table 4:** Summary of frequency-domain features.

Frequency-Domain Features	Authors	Experience (Median Age)	N	Stress		Rest	
				Mean	SD	Mean	SD
LF (ms2)	Klein et al., 2010 [62]	Expert (46)	10	341.75	144.7	533.18	280.52
	Rieger et al., 2014 [53]	Expert (34.5)	12	2358.7	1599	3817.5	2189.52
	Rieger et al., 2014 [53] *	Expert (39)	7	749.75	496.25	792.5	328.5
HF (ms2)	Klein et al., 2010 [62]	Expert (46)	10	102.82	54.32	110.7	68
	Rieger et al., 2014 [53]	Expert (34.5)	12	231.5	133.5	1510.75	785.25
	Rieger et al., 2014 [53] *	Expert (39)	7	122.75	70.25	655.75	241.5
LF/HF (–)	Klein et al., 2010 [62]	Expert (46)	10	4.35	1.5	6.42	3.42
	Jones et al., 2015 [58]	Non-expert (N/A)	16	11.42	4.64	4.02	2.28
	Weenk et al., 2018 [56]	Expert and non-expert (N/A)	20	6.18	3.04	3.97	2.5

* Rieger et al. split his participants into stressed and non-stressed and produced a report on that basis.

## Data Availability

No new data were created or analysed in this study. Data sharing is not applicable to this article.

## Supplementary Materials

The following supporting information can be downloaded at: https://www.mdpi.com/article/10.3390/healthcare14040484/s1, Supplementary File S1: Full used string and full detailed tables; Supplementary File S2: Full information about included studies; Supplementary File S3: MMAT appraisal tool; Supplementary File S4: Inclusion/exclusion file; Supplementary File S5: PRISMA 2020 Checklist. All of the included studies are in the Supplementary Materials, which includes Klein et al., Heemskerk et al., Rieger et al., Jones et al., Weenk et al., Grantcharov et al., Cap et al., Kwon et al., and Haffar et al. [45,53,55,56,57,58,59,62,63].

## Abbreviations

The following abbreviations are used in this manuscript:

HRV	Heart Rate Variability
WHO	World Health Organisation
ANS	Autonomic Nervous System
BMA	British Medical Association
STAI	State–Trait Anxiety Inventory
AMSTAR	Assessing the Methodological Quality of Systematic Reviews
PRISMA	Preferred Reporting Items for Systematic Reviews and Meta-Analyses
PICO	Patient/Problem, Intervention, Comparison, and Outcome
MMAT	The Mixed Methods Appraisal Tool
MD	Mean Difference
CI	Confidence Interval
P	*p*-value
