# Supplementary material for: A Systematic Review and Meta-Analysis of Heart Rate Variability in Assessing Surgeons’ Stress Levels
Source: Healthcare (Basel). 2026 Feb 13;14(4):484. doi: 10.3390/healthcare14040484 (PMC12940869; doi:10.3390/healthcare14040484)
Supplement: Supplementary file 1 [file healthcare-14-00484-s001.zip › S1_File.pdf]

Supplementary Tables

Search strings and results

**Database: PUBMED**			
**N**	**Keywords**	**Strings**	**Results (N)**
#1	Mental stress	mental stress[Title/Abstract] OR sympathetic nerve activity[Title/Abstract] OR mental health[Title/Abstract] OR emotional stress[Title/Abstract] OR mental fatigue[Title/Abstract] OR occupational stress[Title/Abstract] OR physiological stress[Title/Abstract] OR emotional assessment[Title/Abstract] OR ergonomics[Title/Abstract] OR Central Fatigue[Title/Abstract] OR Peripheral Fatigue[Title/Abstract] OR Physical fatigue[Title/Abstract] OR muscle fatigue[Title/Abstract] OR stress[Title/Abstract] OR fatigue[Title/Abstract]	1212251
#2	Continuous monitoring	wearable device[Title/Abstract] OR continuous monitoring[Title/Abstract] OR ECG[Title/Abstract] OR HRV[Title/Abstract] OR detectable indicator[Title/Abstract] OR flexible detector[Title/Abstract] OR wearable sensor[Title/Abstract] OR wireless technology[Title/Abstract] OR remote sensing technology[Title/Abstract] OR vital signs[Title/Abstract] OR telemonitoring[Title/Abstract] OR digital technology[Title/Abstract] OR wearable technology[Title/Abstract] OR e-health[Title/Abstract] OR wearable electronic device[Title/Abstract] OR body sensor[Title/Abstract] OR wireless sensor[Title/Abstract] OR smart sensor[Title/Abstract] OR activity tracker[Title/Abstract] OR mobile health[Title/Abstract] OR wearable healthcare systems[Title/Abstract] OR Heart Rate Variability[Title/Abstract]	141253
#3	Surgery	surgery[Title/Abstract] OR Surgical robotics[Title/Abstract] OR Robot-assisted surgery[Title/Abstract] OR Da Vinci robot[Title/Abstract] OR Minimal invasive surgery[Title/Abstract] OR Robot-assisted[Title/Abstract] OR Laparoscopic surgery[Title/Abstract] OR Robotic-assisted laparoscopy[Title/Abstract] OR operation theatre[Title/Abstract] OR surgeon[Title/Abstract] OR operation room[Title/Abstract] OR robotic surgery[Title/Abstract]	1486284
#1 AND #2 AND #3			513
#1 AND #2 And #3 and from 2002-2022, English, Full Text			326
**Database: SCOPUS**			
#1	Mantal stress	TITLE-ABS (mental AND stress) OR (sympathetic AND nerve AND activity) OR (mental AND health) OR (emotional AND stress) OR (mental AND fatigue) OR (occupational AND stress) OR (physiological AND stress) OR (emotional AND assessment) OR (ergonomics) OR (central AND fatigue) OR (peripheral AND fatigue) OR (physical AND fatigue) OR (muscle AND fatigue) OR (stress) OR (fatigue)	9,340,536
#2	Continuous monitoring	TITLE-ABS (wearable AND device) OR (continuous AND monitoring) OR (ecg) OR (hrv) OR (detectable AND indicator) OR (flexible AND detector) OR (wearable AND sensor) OR (wireless AND technology) OR (remote AND sensing AND technology) OR (vital AND signs) OR (telemonitoring) OR (digital AND technology) OR (wearable AND technology) OR (e-health) OR (wearable AND electronic AND device) OR (body AND sensor) OR (wireless AND sensor) OR (smart AND sensor) OR (activity AND tracker) OR (mobile AND health) OR (wearable AND health AND care AND systems) OR (heart AND rate AND variability)	5,628,315
#3	Surgery	TITLE-ABS (surgery OR (surgical AND robotics) OR (robot-assisted AND surgery) OR (da AND vinci AND robot) OR (minimal AND invasive AND surgery) OR (robot-assisted) OR (laparoscopic AND surgery) OR (robotic-assisted AND laparoscopy) OR (operation AND theatre) OR surgeon OR (operation AND room) OR (robotic AND surgery))	1,862,742
#1 AND #2 AND #3			15,207
#1 AND #2 AND #3 from 2002-2022, English			1,496
**Database: Web of science**			
#1	Mental stress	((AB=(mental stress OR sympathetic nerve activity OR mental health OR emotional stress OR mental fatigue OR occupational stress OR physiological stress OR emotional assessment OR ergonomics OR Central Fatigue OR Peripheral Fatigue OR Physical fatigue OR muscle fatigue OR stress OR fatigue)) AND TI=(mental	504,899

		stress OR sympathetic nerve activity OR mental health OR emotional stress OR mental fatigue OR occupational stress OR physiological stress OR emotional assessment OR ergonomics OR Central Fatigue OR Peripheral Fatigue OR stress OR fatigue OR Physical fatigue OR muscle fatigue OR stress OR fatigue))	
#2	Continuous monitoring	((TI=(wearable device OR continuous monitoring OR ECG OR HRV OR detectable indicator OR flexible detector OR wearable sensor OR wireless technology OR remote sensing technology OR vital signs OR telemonitoring OR digital technology OR wearable technology OR e-health OR wearable electronic device OR body sensor OR wireless sensor OR smart sensor OR activity tracker OR mobile health OR wearable healthcare systems OR Heart Rate Variability)) AND AB=(wearable device OR continuous monitoring OR ECG OR HRV OR detectable indicator OR flexible detector OR wearable sensor OR wireless technology OR remote sensing technology OR vital signs OR telemonitoring OR digital technology OR wearable technology OR e-health OR wearable electronic device OR body sensor OR wireless sensor OR smart sensor OR activity tracker OR mobile health OR wearable healthcare systems OR Heart Rate Variability))	97,589
#3	Surgery	(TI=(surgery OR Surgical robotics OR Robot-assisted surgery OR Da Vinci robot OR Minimal invasive surgery OR Robot-assisted OR Laparoscopic surgery OR Robotic-assisted laparoscopy OR operation theatre OR surgeon OR operation room OR robotic surgery)) AND AB=(surgery OR Surgical robotics OR Robot-assisted surgery OR Da Vinci robot OR Minimal invasive surgery OR Robot-assisted OR Laparoscopic surgery OR Robotic-assisted laparoscopy OR operation theatre OR surgeon OR operation room OR robotic surgery)	216,244
#1 AND #2 AND #3			11
#1 AND #2 AND #3 AND from 2002 to 2022, English			10

Updated search strings and results

**Database: PUBMED**			
**N**	**Keywords**	**Strings**	**Results (N)**
#1	Mental stress	mental stress[Title/Abstract] OR sympathetic nerve activity[Title/Abstract] OR mental health[Title/Abstract] OR emotional stress[Title/Abstract] OR mental fatigue[Title/Abstract] OR occupational stress[Title/Abstract] OR physiological stress[Title/Abstract] OR emotional assessment[Title/Abstract] OR ergonomics[Title/Abstract] OR Central Fatigue[Title/Abstract] OR Peripheral Fatigue[Title/Abstract] OR Physical fatigue[Title/Abstract] OR muscle fatigue[Title/Abstract] OR stress[Title/Abstract] OR fatigue[Title/Abstract]	1,367,030
#2	Continuous monitoring	wearable device[Title/Abstract] OR continuous monitoring[Title/Abstract] OR ECG[Title/Abstract] OR HRV[Title/Abstract] OR detectable indicator[Title/Abstract] OR flexible detector[Title/Abstract] OR wearable sensor[Title/Abstract] OR wireless technology[Title/Abstract] OR remote sensing technology[Title/Abstract] OR vital signs[Title/Abstract] OR telemonitoring[Title/Abstract] OR digital technology[Title/Abstract] OR wearable technology[Title/Abstract] OR e-health[Title/Abstract] OR wearable electronic device[Title/Abstract] OR body sensor[Title/Abstract] OR wireless sensor[Title/Abstract] OR smart sensor[Title/Abstract] OR activity tracker[Title/Abstract] OR mobile health[Title/Abstract] OR wearable healthcare systems[Title/Abstract] OR Heart Rate Variability[Title/Abstract]	157,754
#3	Surgery	surgery[Title/Abstract] OR Surgical robotics[Title/Abstract] OR Robot-assisted surgery[Title/Abstract] OR Da Vinci robot[Title/Abstract] OR Minimal invasive surgery[Title/Abstract] OR Robot-assisted[Title/Abstract] OR Laparoscopic surgery[Title/Abstract] OR Robotic-assisted laparoscopy[Title/Abstract] OR operation theatre[Title/Abstract] OR surgeon[Title/Abstract] OR operation room[Title/Abstract] OR robotic surgery[Title/Abstract]	1,613,923

#1 AND #2 AND #3			580
#1 AND #2 And #3 and from 2022-2024, English, Full Text			99
**Database: SCOPUS**			
#1	Mantal stress	TITLE-ABS (mental AND stress) OR (sympathetic AND nerve AND activity) OR (mental AND health) OR (emotional AND stress) OR (mental AND fatigue) OR (occupational AND stress) OR (physiological AND stress) OR (emotional AND assessment) OR (ergonomics) OR (central AND fatigue) OR (peripheral AND fatigue) OR (physical AND fatigue) OR (muscle AND fatigue) OR (stress) OR (fatigue)	10,657,866
#2	Continuous monitoring	TITLE-ABS (wearable AND device) OR (continuous AND monitoring) OR (ecg) OR (hrv) OR (detectable AND indicator) OR (flexible AND detector) OR (wearable AND sensor) OR (wireless AND technology) OR (remote AND sensing AND technology) OR (vital AND signs) OR (telemonitoring) OR (digital AND technology) OR (wearable AND technology) OR (e-health) OR (wearable AND electronic AND device) OR (body AND sensor) OR (wireless AND sensor) OR (smart AND sensor) OR (activity AND tracker) OR (mobile AND health) OR (wearable AND health AND care AND systems) OR (heart AND rate AND variability)	6,884,751
#3	Surgery	TITLE-ABS (surgery OR (surgical AND robotics) OR (robot-assisted AND surgery) OR (da AND vinci AND robot) OR (minimal AND invasive AND surgery) OR (robot-assisted) OR (laparoscopic AND surgery) OR (robotic-assisted AND laparoscopy) OR (operation AND theatre) OR surgeon OR (operation AND room) OR (robotic AND surgery))	2,043,847
#1 AND #2 AND #3			518
#1 AND #2 AND #3 from 2002-2022, English			47
**Database: Web of science**			
#1	Mental stress	((AB=(mental stress OR sympathetic nerve activity OR mental health OR emotional stress OR mental fatigue OR occupational stress OR physiological stress OR emotional assessment OR ergonomics OR Central Fatigue OR Peripheral Fatigue OR Physical fatigue OR muscle fatigue OR stress OR fatigue)) AND TI=(mental stress OR sympathetic nerve activity OR mental health OR emotional stress OR mental fatigue OR occupational stress OR physiological stress OR emotional assessment OR ergonomics OR Central Fatigue OR Peripheral Fatigue OR stress OR fatigue OR Physical fatigue OR muscle fatigue OR stress OR fatigue))	580,196
#2	Continuous monitoring	((TI=(wearable device OR continuous monitoring OR ECG OR HRV OR detectable indicator OR flexible detector OR wearable sensor OR wireless technology OR remote sensing technology OR vital signs OR telemonitoring OR digital technology OR wearable technology OR e-health OR wearable electronic device OR body sensor OR wireless sensor OR smart sensor OR activity tracker OR mobile health OR wearable healthcare systems OR Heart Rate Variability)) AND AB=(wearable device OR continuous monitoring OR ECG OR HRV OR detectable indicator OR flexible detector OR wearable sensor OR wireless technology OR remote sensing technology OR vital signs OR telemonitoring OR digital technology OR wearable technology OR e-health OR wearable electronic device OR body sensor OR wireless sensor OR smart sensor OR activity tracker OR mobile health OR wearable healthcare systems OR Heart Rate Variability))	111,432
#3	Surgery	(TI=(surgery OR Surgical robotics OR Robot-assisted surgery OR Da Vinci robot OR Minimal invasive surgery OR Robot-assisted OR Laparoscopic surgery OR Robotic-assisted laparoscopy OR operation theatre OR surgeon OR operation room OR robotic surgery)) AND AB=(surgery OR Surgical robotics OR Robot-assisted surgery OR Da Vinci robot OR Minimal invasive surgery OR Robot-assisted OR Laparoscopic surgery OR Robotic-assisted laparoscopy OR operation theatre OR surgeon OR operation room OR robotic surgery)	253,452
#1 AND #2 AND #3			13
#1 AND #2 AND #3 AND from 2002 to 2022, English			3

HEART RATE

paper	Age	N sample size	Experience (Note)	Mean (HR)	SD (HR)
Weenk- 2018	N/A	20	Baseline/general	68.335	9.01
Weenk- 2018	N/A	20 (63 procedure)	Surgery/ general	89.52	14.515
Weenk- 2018, a	N/A	11	Baseline/ Male	67.62	9.01
Weenk- 2018, a	N/A	11	Surgery/ Male	85.275	11.025
Weenk- 2018, a	N/A	9	Baseline/ Female	69.045	3.99
Weenk- 2018, a	N/A	9	Surgery/ Female	94.705	12.85
Weenk-2018, b	Mean 46.2	5	Consultant (during surgery)	77.3725	8.7125
Weenk-2018, b	Mean 35.4	7	Fellows and senior residents (during surgery)	94.655	12.3
Weenk-2018, b	Mean 32.2	8	Junior residents (during surgery)	89.15	8.89
Klein- 2010	Median 46	10	Surgery (expert) (standard OR)	89.05	14.1
Klein- 2010	Median 46	10	Surgery (expert) (modern OR)	84.675	14.375
Rieger- 2014	Median 34.5	12	Baseline (expert) (non-stressed)	58.15	8.7
Rieger- 2014	Median 34.5	12	Surgery (expert) (non-stressed)	84.3	10.65
Rieger- 2014, a	Median 39	7	Baseline (expert)(stressed)	64.5	9.3
Rieger- 2014, a	Median 39	7	Surgery (expert) (stressed)	106.6	15.95
Cap-2021	Mean 42.5	5	Baseline (Mixed)	108.875	1.625
Cap-2021	Mean 42.5	5	Surgery (mixed)	88.75	10.75
Jones-2015	N/A	6(16 procedure)	Baseline (non-expert)	67	14.89
Jones-2015	N/A	6(16procedure)	Surgery (non-expert)	88	20.49
Kwon- 2021	Mean 42	8	Baseline (non-expert)	73.1	5.8
Heemskerk-2014	N/A	2	Baseline(Robotic) (expert)	84.6	0
Heemskerk-2014	N/A	2	Baseline (Labaroscopic) (expert)	84.9	0
Heemskerk-2014,a	N/A	2	Surgery (Trocar placement) (Robotic) (expert)	82.7	0

Heemskerk-2014,a	N/A	2	Surgery (Trocar placement) (laparoscopic) (expert)	89.2	0
Heemskerk-2014, b	N/A	2	Surgery(Dissection calot) (robotic) (expert)	78.7	0
Heemskerk-2014, b	N/A	2	Surgery(Dissection calot) (laparoscopic) (expert)	92.8	0
Heemskerk-2014, c	N/A	2	Surgery (clip and cut) (robotic) (expert)	75.7	0
Heemskerk-2014, c	N/A	2	Surgery (clip and cut) (laparoscopic) (expert)	97.2	0
Heemskerk-2014, d	N/A	2	Surgery (Dissection gallbladder) (robotic) (expert)	75.3	0
Heemskerk-2014, d	N/A	2	Surgery (Dissection gallbladder) (laparoscopic) (expert)	96.2	0
Heemskerk-2014,e	N/A	2	Surgery (Start removal) (robotic) (expert)	76.6	0
Heemskerk-2014,e	N/A	2	Surgery (Start removal) (laparoscopic) (expert)	95.6	0
Heemskerk-2014, f	N/A	2	Surgery (closure) (robotic) (expert)	79.3	0
Heemslerl-2014,f	N/A	2	Surgery (closure) (laparoscopic) (expert)	91.3	0
Haffar- 2022	N/A	1(20 procedure)	surgery (cTKA) (expert)	90.1	0
Haffar- 2022	N/A	1 (20 procedure	surgery (rTKA) (not-expert)	81.5	0

Table 1: Heart rate readings in detail as reported

SDNN

paper	Age	N sample size	Note	Mean (SDNN)	SD (SDNN)
Weenk- 2018	N/A	20	Baseline\general	81.03	27.86
Weenk- 2018	N/A	63	Surgery\ general	51	12.08
Weenk- 2018, a	N/A	11	Baseline	87.94	3.31
Weenk- 2018, a	N/A	11	Surgeery	54.69	11.66
Weenk- 2018, a	N/A	9	Baseline	72.57	23.41
Weenk- 2018, a	N/A	9	Surgery	46.67	11.26
Weenk-2018, b	Mean 46.2	5	Consultants (during surgery)	59.47	13.82
Weenk-2018, b	Mean 35.4	7	Fellows and senior residents (during surgery)	48.05	10.38

Weenk-2018, b	Mean 32.2	8	Junior residents (during surgery)	50.36	11.68
Klein- 2010	Median 46	10	Surgery (standered OR)	48.225	22.275
Klein- 2010	Median 46	10	Surgery (modern OR)	39.075	16.375
Rieger- 2014	Median 34.5	12	Baseline(non-stressed)	60.025	19.575
Rieger- 2014	Median 34.5	12	Surgery(non-stressed)	43.675	14.075
Rieger- 2014, a	Median 39	7	Baseline (stressed)	41.8	1.85
Rieger- 2014, a	Median 39	7	Surgery(stressed)	29.05	11.5
Cap-2021	Mean 42.5	5	Surgery	54.2	13.55
Grantcharov-2019	N/A	1	Baseline	40.4	3.35
Grantcharov-2019	N/A	1	Surgery	38.16	3.75

Table 2: SDNN readings in detail as reported

pNN50

paper	Age	N sample size	Note	Mean (pNN50)	SD (pNN50)
Klein- 2010	Median 46	10	Surgery (standered OR)	7.9	7.15
Klein- 2010	Median 46	10	Surgery (modern OR)	4.525	3.425
Rieger- 2014	Mean 34.75	12	Baseline(non-stressed)	40	16.8
Rieger- 2014	Mean 34.75	12	Surgery(non-stressed)	7.15	4.7
Rieger- 2014, a	Mean 41.5	7	Baseline (stressed)	28.475	3.425
Rieger- 2014, a	Mean 41.5	7	Surgery(stressed)	5.15	3.7

paper	Age	N sample size	Note	Mean (pNN50)	SD (pNN50)
Klein- 2010	Median 46	10	Surgery (standered OR)	7.9	7.15
Klein- 2010	Median 46	10	Surgery (modern OR)	4.525	3.425

Rieger- 2014	Mean 34.75	12	Baseline(non-stressed)	40	16.8
Rieger- 2014	Mean 34.75	12	Surgery(non-stressed)	7.15	4.7
Rieger- 2014, a	Mean 41.5	7	Baseline (stressed)	28.475	3.425
Rieger- 2014, a	Mean 41.5	7	Surgery(stressed)	5.15	3.7

Table 3: Pnn50 readings in detail as reported

RMSSD

paper	Age	N sample size	Note	Mean (RMSSD)	SD (RMSSD)
Weenk- 2018	N/A	20	Baseline\general (control)	57.91	27.425
Weenk- 2018	N/A	63	Surgery\ general (expermintal)	28.5875	10.2675
Weenk- 2018, a	N/A	11	Baseline	62.415	27.425
Weenk- 2018, a	N/A	11	Surgeery	31.475	9.505
Weenk- 2018, a	N/A	9	Baseline	40.7325	13.8025
Weenk- 2018, a	N/A	9	Surgery	24.4575	7.3875
Weenk-2018, b	mean 46.2	5	Consultants (during surgery)	31.03	3.88
Weenk-2018, b	mean 35.4	7	Fellows senior residents (during surgery)	27.1125	10.2675
Weenk-2018, b	mean 32.2	8	Junior residents (during surgery)	27.6175	9.5025
Klein- 2010	Median 46	10	Surgery (standered OR) (control)	22.95	13.5
Klein- 2010	Median 46	10	Surgery (modern OR) (expermintal)	20.675	6.725
Rieger- 2014	Median 34.5	12	Baseline(non-stressed) (control)	71.9	26.1
Rieger- 2014	Median 34.5	12	Surgery(non-stressed) (expermintal)	25.85	8.15
Rieger- 2014, a	Median 39	7	Baseline (stressed) (control)	46.75	7.6
Rieger- 2014, a	Median 39	7	Surgery(stressed) (expermintal)	20.05	7.65
Grantcharov-2019	N/A	1	Baseline (control)	25.8	7.4
Grantcharov-2019	N/A	1	Surgery (expermintal)	24.67	1.8

Kwon- 2021	Mean 42	8	Baseline	31.4	4.3
Haffar- 2022	N/A	1(20 procedure)	surgery (cTKA) (ocntrol)	12.2	0
Haffar- 2022	N/A	1 (20 procedure	surgery (rTKA) (expermintal)	14.4	0

Table 4: RMSSD readings in detail as reported

LF

paper	Age	N sample size	Note	Mean (LF)	SD (LF)
Klein- 2010	Median 46	10	Surgery (standered OR)	533.175	280.525
Klein- 2010	Median 46	10	Surgery (modern OR)	341.75	144.7
Rieger- 2014	Median 34.5	12	Baseline(non-stressed)	3817.5	2189.25
Rieger- 2014	Median 34.5	12	Surgery(non-stressed)	2358.75	1599
Rieger- 2014, a	Median 39	7	Baseline (stressed)	792.5	328.5
Rieger- 2014, a	Median 39	7	Surgery(stressed)	749.75	496.25
Heemskerk- 2014	N/A	2	Baseline(Robotic)	1007.5	535
Heemskerk- 2014	N/A	2	Baseline (Labaroscopic)	1251.75	596.25
Heemskerk- 2014,a	N/A	2	Surgery (Trocar placement)(Robotic)	1104.5	336
Heemskerk- 2014,a	N/A	2	Surgery (Trocar placement)(laparoscopic)	1093.75	586.25
Heemskerk- 2014, b	N/A	2	Surgery(Dissection calot) (robotic)	1475.75	843.25
Heemskerk- 2014, b	N/A	2	Surgery(Dissection calot) (laparoscopic)	484.25	149.75
Heemskerk- 2014, c	N/A	2	Surgery (clip and cut) (robotic)	1528.25	1147.75
Heemskerk- 2014, c	N/A	2	Surgery (clip and cut)(laparoscopic)	609.75	214.75
Heemskerk- 2014, d	N/A	2	Surgery (Dissection gallbladder) (robotic)	1302	885

Heemskerk-2014, d	N/A	2	Surgery (Dissection gallbladder) (lararoscopic)	550.5	235.5
Heemskerk-2014,e	N/A	2	Surgery (Start removal) (robotic)	1073.5	487.5
Heemskerk-2014,e	N/A	2	Surgery (Start removal) (laparoscopic)	549.5	232
Heemskerk-2014, f	N/A	2	Surgery (closure) (robotic)	2493.25	1168.25
Heemslerl-2014,f	N/A	2	Surgery (closure) (laparoscopic)	622.25	247.75

Table 5: LF readings in detail as reported

HF

paper	Age	N sample size	Note	Mean (HF)	SD (HF)
Klein- 2010	Median 46	10	Surgery (standered OR)	110.7	68
Klein- 2010	Median 46	10	Surgery (modern OR)	102.825	54.325
Rieger- 2014	Median 34.5	12	Baseline(non-stressed)	1510.75	785.25
Rieger- 2014	Median 34.5	12	Surgery(non-stressed)	231.5	133.5
Rieger- 2014, a	Median 39	7	Baseline (stressed)	655.75	241.25
Rieger- 2014, a	Median 39	7	Surgery(stressed)	122.75	70.25
Heemskerk-2014	N/A	2	Baseline(Robotic)	861.25	705.75
Heemskerk-2014,	N/A	2	Baseline (Labaroscopic)	450.25	239.25
Heemskerk-2014,a	N/A	2	Surgery (Trocar placement)(Robotic)	1033.75	726.25
Heemskerk-2014,a	N/A	2	Surgery (Trocar placement)(laparoscopic)	454	324.5
Heemskerk-2014, b	N/A	2	Surgery(Dissection calot) (robotic)	598	367.5
Heemskerk-2014, b	N/A	2	Surgery(Dissection calot) (laparoscopic)	228.75	82.25
Heemskerk-2014, c	N/A	2	Surgery (clip and cut) (robotic)	1764.25	1463.25
Heemskerk-2014, c	N/A	2	Surgery (clip and cut) (laparoscopic)	191.25	80.75

Heemskerk-2014, d	N/A	2	Surgery (Dissection gallbladder) (robotic)	1089.25	726.25
Heemskerk-2014, d	N/A	2	Surgery (Dissection gallbladder) (lararoscopic)	221.5	109.5
Heemskerk-2014,e	N/A	2	Surgery (Start removal) (robotic)	1591	1209
Heemskerk-2014,e	N/A	2	Surgery (Start removal) (laparoscopic)	204.75	96.25
Heemskerk-2014, f	N/A	2	Surgery (closure) (robotic)	1144.5	805
Heemslerk-2014,f	N/A	2	Surgery (closure) (laparoscopic)	157	43

Table 6: HF readings in detail as reported

LF/HF

paper	Age	Note	Mean (LF/HF)	SD (LF/HF)
Weenk- 2018	N/A	Baseline\general	3.97	2.5
Weenk- 2018	N/A	Surgery\ general	6.18	3.04
Weenk-2018, a	N/A	Baseline	3.95	2.76
Weenk-2018, a	N/A	Surgeery	6.22	3.16
Weenk-2018, a	N/A	Baseline	4	2.31
Weenk-2018, a	N/A	Surgery	6.13	2.96
Weenk-2018, b	mean 46.2	Consultants (during surgery)	5.6	1.61
Weenk-2018, b	mean 35.4	Fellows and senior residents (during surgery)	6.59	3.41
Weenk-2018, b	mean 32.2	Junior residents (during surgery)	6.02	3.16
Klein- 2010	Median 46	Surgery (standered OR)	6.425	3.425
Klein- 2010	Median 46	Surgery (modern OR)	4.35	1.5
Jones-2015	N/A	Baseline	4.02	2.283
Jones-2015	N/A	Surgery	11.42	4.636
Kwon- 2021	mean 42	Baseline	0.8125	0.6
Heemskerk-2014	N/A	Baseline(Robotic)	3.635	2.11
Heemskerk-2014	N/A	Baseline (Labaroscopic)	3.6125	1.4925
Heemskerk-2014,a	N/A	Surgery (Trocar placement)(Robotic)	2.63	1.36

Heemskerk-2014,a	N/A	Surgery (Trocar placement)(laparoscopic)	4.3825	1.9925
Heemskerk-2014, b	N/A	Surgery(Dissection calot) (robotic)	2.46	1.005
Heemskerk-2014, b	N/A	Surgery(Dissection calot) (laparoscopic)	2.92	1.25
Heemskerk-2014, c	N/A	Surgery (clip and cut) (robotic)	1.235	0.475
Heemskerk-2014, c	N/A	Surgery (clip and cut) (laparoscopic)	3.305	0.985
Heemskerk-2014, d	N/A	Surgery (Dissection gallbladder) (robotic)	1.6825	0.7575
Heemskerk-2014, d	N/A	Surgery (Dissection gallbladder) (lararoscopic)	2.7925	0.8475
Heemskerk-2014,e	N/A	Surgery (Start removal) (robotic)	1.4925	0.5575
Heemskerk-2014,e	N/A	Surgery (Start removal) (laparoscopic)	3.815	1.79
Heemskerk-2014, f	N/A	Surgery (closure) (robotic)	3.33	1.13
Heemslerl-2014,f	N/A	Surgery (closure) (laparoscopic)	3.985	1.47

Table 7: LF/HF readings in detail as reported
